# Biological and Technical Challenges in Unraveling the Role of N-Glycans in Immune Receptor Regulation

**DOI:** 10.3389/fchem.2020.00055

**Published:** 2020-02-05

**Authors:** Paola de Haas, Wiljan J. A. J. Hendriks, Dirk J. Lefeber, Alessandra Cambi

**Affiliations:** ^1^Department of Cell Biology, Radboud Institute for Molecular Life Sciences, Radboud University Medical Center, Nijmegen, Netherlands; ^2^Department of Laboratory Medicine, Translational Metabolic Laboratory, Radboud Institute for Molecular Life Sciences, Radboud University Medical Center, Nijmegen, Netherlands; ^3^Department of Neurology, Donders Institute for Brain, Cognition and Behaviour, Radboud University Medical Center, Nijmegen, Netherlands

**Keywords:** membrane receptor, glycocalyx, N-glycans, immune receptor, glycoprotein, galectin, cell membrane, protein glycoforms

## Abstract

N-glycosylation of membrane receptors is important for a wide variety of cellular processes. In the immune system, loss or alteration of receptor glycosylation can affect pathogen recognition, cell-cell interaction, and activation as well as migration. This is not only due to aberrant folding of the receptor, but also to altered lateral mobility or aggregation capacity. Despite increasing evidence of their biological relevance, glycosylation-dependent mechanisms of receptor regulation are hard to dissect at the molecular level. This is due to the intrinsic complexity of the glycosylation process and high diversity of glycan structures combined with the technical limitations of the current experimental tools. It is still challenging to precisely determine the localization and site-occupancy of glycosylation sites, glycan micro- and macro-heterogeneity at the individual receptor level as well as the biological function and specific interactome of receptor glycoforms. In addition, the tools available to manipulate N-glycans of a specific receptor are limited. Significant progress has however been made thanks to innovative approaches such as glycoproteomics, metabolic engineering, or chemoenzymatic labeling. By discussing examples of immune receptors involved in pathogen recognition, migration, antigen presentation, and cell signaling, this Mini Review will focus on the biological importance of N-glycosylation for receptor functions and highlight the technical challenges for examination and manipulation of receptor N-glycans.

## Introduction

Protein glycosylation, the enzymatic addition of glycans to amino acid side chains, is the most common post-translational modification. It is exerted by an intricate set of enzymes that prunes and grafts the carbohydrate moieties on proteins traveling the endoplasmic reticulum (ER)—Golgi apparatus secretory route toward the cell surface (Ohtsubo and Marth, 2006).

Glycans are composed of different monosaccharides, that can be covalently bound via alpha or beta linkages, rendering a wide range of glycan structures. Competition and dynamics of glycosylation enzymes lead to varying glycan structures not only on different proteins but also within the same protein, a phenomenon called glycan microheterogeneity. Also, one protein can have multiple glycosylation sites that may be partially occupied, reflecting variations in enzymes, substrate, and protein acceptor fluxes (Zacchi and Schulz, 2016), resulting in glycan macroheterogeneity. Glycan heterogeneity occurs among different tissues (Medzihradszky et al., 2015), biomarks developmental and activation stages (Clark and Baum, 2012), and correlates to aging and disease processes (Kristic et al., 2014; Reily et al., 2019).

Two major types of protein glycosylation are discerned: N-glycosylation, the addition of glycan chains on the amide nitrogen of asparagine residues in the ER, and O-glycosylation, the addition of glycan chains to the oxygen atom of serine/threonine residues in the Golgi. For many membrane proteins, removal of N-glycosylation sites leads to intracellular retention, degradation, and reduced membrane expression (Barbosa et al., 1987; Fischer et al., 2017). Moreover, after arrival on the cell surface, the stability and functionality of glycoproteins remain dependent on their glycosylation pattern (Dennis et al., 2009a,b; Skropeta, 2009). This partly relates to the existence of membrane-associated β-galactoside-binding lectins, known as galectins, which bind and crosslink different glycoproteins (Nabi et al., 2015). Since galectins have multiple binding sites and form multimers, they enable the formation of galectin lattices: an intricate network of glycoproteins and glycan-binding proteins, which shapes and regulates cell surface subdomains of clustered macromolecules and ultimately influences cell functions (Lajoie et al., 2009).

Aberrant protein glycosylation impacts viability and functionality of cells and organisms. This is underlined by the Congenital Disorders of Glycosylation, where defects in the synthesis or processing of glycans affect glycoprotein activities and cause a large variety of systemic symptoms (Peanne et al., 2018). Furthermore, Mkhikian et al. demonstrated that in multiple sclerosis environmental factors and genetic variants of immune receptors and glycosylation enzymes collectively dysregulate the N-glycosylation pathway (Mkhikian et al., 2011). Finally, malignant transformation is notoriously associated with altered glycosylation (Marsico et al., 2018). Despite its clear biomedical relevance, addressing the involvement of glycosylation in protein activity is challenging due to the complexity of the glycosylation process and the relatively limited experimental tools available.

**Figure 1:**
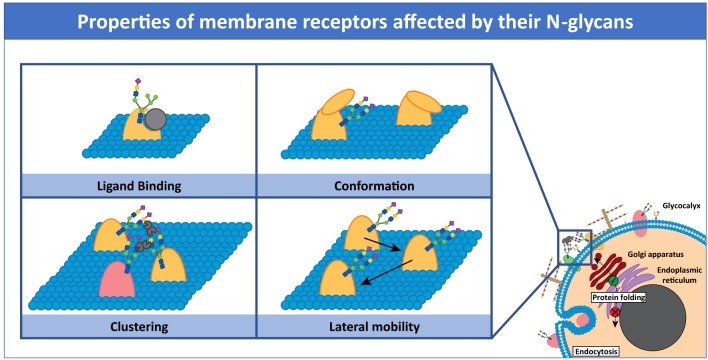
Properties of membrane receptors influenced by N-glycans. The presence or absence of N-glycans on receptor molecules can modulate receptor function by affecting not only folding but also ligand binding, conformation, clustering, and lateral mobility. Glycan-crosslinking proteins, such as galectins, can influence these properties.

Recent reviews highlight the importance of protein-glycan interactions in immunity (Marth and Grewal, 2008; Van Kooyk and Rabinovich, 2008; Zhou et al., 2018). In this review, we highlight key examples of how N-glycosylation of membrane immune receptors influences receptor properties, such as lateral interactions with neighboring molecules, clustering and diffusion behavior, all modulating receptor function (Figure 1). We also discuss tools and approaches for detection and manipulation of membrane receptor glycosylation, emphasizing current challenges and opportunities (Table 1).

**Table 1:** Overview of the techniques to study membrane receptor glycosylation: applications and limitations.

**Tools for studying membrane receptor glycosylation**				
**Method**	**Working principle**	**Applications**	**Limitations**	**References**
**Quantitative and qualitive measurement of receptor glycosylation**				
SDS-PAGE and glycan-cleaving enzymes	Separating glycoforms by differences in molecular weight, using cell lysates treated with or without glycan-cleaving enzymes.	Quick and easy way to observe different glycoforms and roughly assess localization: membrane vs. cytosol.	No detailed information on glycan structure. Glycoforms with small or no differences in molecular weight cannot be separated.	Freeze and Kranz, 2010
Lectin blot	Separating glycoforms by differences in molecular weight and staining with glycan binding lectins.	Analyze different glycoforms in cell lysates and provides insights regarding glycan composition.	Low binding affinity and specificity of lectins. No detailed information on glycan structure.	Cao et al., 2013
Chemoenzymatic labeling	Building a monosaccharide analog into the glycan chain to introduce a chemical modification compatible with click chemistry reactions.	Specific glycan structures can be labeled on live cells or cell lysates.	No detailed information on glycan structure or site occupancy. Labeling is not specific for the protein of interest.	Lopez Aguilar et al., 2017
Lectin and antibody labeling	Visualizing specific glycan structures by lectin or antibody staining.	Labeling specific glycan structures for microscopy or flow cytometry.	Low binding affinity and specificity of lectins. Labeling is not specific for the protein of interest.	Tommasone et al., 2019
*In situ* proximity ligation assay	Antibodies conjugated to oligonucleotides can only ligate and be visualized when the antibody targets are in close proximity.	Provides information on the location of glycoforms within the cell and on the cell membrane.	No detailed information on glycan structure. Distance between the detected entities can be up to 40 nm, sometimes complicating data interpretation.	Soderberg et al., 2006
Glycomics	Analyzing glycan structure with mass spectrometry-based techniques.	Provides detailed structural information on glycans released from their protein backbone.	No information on the location of the glycosylation site within the protein.	Lauc and Wuhrer, 2017
Glycoproteomics	Analyzing glycopeptides with mass spectrometry-based techniques.	Provides information on the location of the glycosylation site and the glycan structure as well as micro- and macro-heterogeneity.	Glycan fine structures cannot be dissected when using large scale glycopeptide analysis. Complicated data analysis creating many false positive identifications.	Lauc and Wuhrer, 2017; Yang et al., 2017; Narimatsu H. et al., 2018
FRET-based labeling of glycoproteins	Energy transfer from a donor fluorophore to an acceptor fluorophore can only occur when the antibody targets are in close proximity.	Provides information on the cellular location of glycoforms.	No detailed information on glycan structure and site-occupancy.	Lin et al., 2014
**Strategies for manipulation of membrane receptor glycosylation**				
Metabolic inhibitors	Different strategies, examples are sugar analogs, oligosaccharyltransferase inhibitors or inhibitors of glycosyltransferases.	Inhibition of the glycosylation pathway resulting in loss, truncation or modification of glycans.	Unwanted side-effects. Effect not specific for protein of interest.	Wojtowicz et al., 2012
Knockdown or knockout of glycosyltransferases	RNA interference or CRISPR-Cas9 genome editing.	Inhibition of the glycosylation pathway resulting in loss, truncation or modification of glycans.	Need for easy-to-transfect cell-line. Effect not specific for protein of interest.	Stolfa et al., 2016; Narimatsu Y. et al., 2018
Site-directed mutagenesis	Changing the glycosylation consensus sequence by changing the amino acid sequence of the protein and therefore removing the glycosylation site.	Removing one or multiple glycan chains from a specific protein.	Need for easy-to-transfect cell-line. Amino-acid substitution can affect other protein properties besides glycan presence. Modification of glycans is not possible.	Barbosa et al., 1987; Bordo and Argos, 1991; Weber et al., 2004

## Membrane Immune Receptor Glycosylation: Beyond Protein Folding

### Pathogen Recognition

Many membrane receptors involved in pathogen-recognition are glycosylated and their glycans can influence their function. For example, Dendritic Cell Immunoreceptor (DCIR) has one N-glycosylation site inside its carbohydrate-recognition domain. Removing or truncating this glycan increases the affinity for DCIR-binding ligands, but the underlying mechanism is still undefined (Bloem et al., 2013). For other pathogen recognition receptors, more information on N-glycosylation impact is available and will be discussed.

DC-SIGN is a homo-tetramer expressed by human macrophages and dendritic cells (Geijtenbeek et al., 2000) and organizes in nanoclusters at the cell membrane, which are specifically important for binding of virus-size particles (Cambi et al., 2004). Mutagenesis of its single N-glycosylation site (N80A) does not alter the expression levels and overall binding capacity nor nanocluster formation. However, unlike the wild-type receptor, the DC-SIGN-N80A mutant exhibits clathrin-independent internalization of virus particles and reduced adhesion strengthening when binding *Candida albicans* (Torreno-Pina et al., 2014; Te Riet et al., 2017). This could be explained by DC-SIGN-N80A inability to laterally interact with actin-anchored transmembrane glycoproteins like CD44. Wildtype DC-SIGN diffusion pattern—but not that of DC-SIGN-N80A—indeed overlaps with that of CD44, being restricted to membrane areas of high clathrin density (Torreno-Pina et al., 2014). This interaction is possibly regulated by galectins since proteomic studies show colocalization of DC-SIGN, CD44, and Galectin-9 at the phagosome (Buschow et al., 2012), and lactose addition, which competes with binding of extracellular galectins to N-glycan chains, prevents adhesion strengthening during DC-SIGN-pathogen binding (Te Riet et al., 2017).

The pathogen-recognition receptor Dectin-1, which binds fungal β-glucan, is also shown to be dependent on galectin interactions. A N-glycan-dependent close association of Dectin-1 with Galectin-3 is demonstrated on murine macrophage membranes, which is required for the proinflammatory response to pathogenic fungi (Esteban et al., 2011; Leclaire et al., 2018). Removal of Dectin-1 N-glycans lowers surface expression levels, negatively influencing ligand-binding and the collaboration with Toll-like Receptor 2 (TLR2; Kato et al., 2006).

Interactions of TLR2 and TLR4 with Galectin-3 are also documented. Macrophages differently sense pathogenic and non-pathogenic fungi thanks to a TLR2-Galectin-3 association induced by ligands specifically present on the pathogen's surface (Jouault et al., 2006). Galectin-3-dependent TLR4 activation contributes to sustained microglia activation, prolonging the inflammatory response in neuroinflammatory diseases (Esteban et al., 2011; Burguillos et al., 2015). TLR4 has 9 N-linked glycosylation sites and is part of the lipopolysaccharide (LPS) receptor complex together with the glycoproteins CD14 and MD-2. The N-linked glycans of both MD-2 and TLR4 are essential in maintaining the functional integrity of the LPS receptor (Da Silva Correia and Ulevitch, 2002).

### Antigen Presentation

Not only antigen recognition and uptake but also antigen presentation is influenced by N-glycosylation. All major histocompatibility (MHC) glycoprotein family members contain evolutionary highly conserved putative sites for N-glycosylation, suggesting functional relevance (Ryan and Cobb, 2012). Indeed, removal of N-glycans from MHCIa molecules by mutagenesis of glycosylation sites significantly increases protein misfolding thus decreasing surface expression levels (Barbosa et al., 1987). Additionally, the MHC-I glycan chains are critical for its association with calreticulin, which is required to form a stable MHCI-antigenic peptide loading complex that is necessary to elicit a T cell response (Van Der Burg et al., 1996; Wearsch et al., 2011; Zhang et al., 2011).

Removal of the complex N-glycans on MHCII molecules, using inhibitors catanospermine and kifunensine or by knockout of the glycosyltransferase gene *MGAT2*, reduces binding and presentation of glycoantigens, but not of peptide antigens, and reduces T cell activation (Ryan et al., 2011). Authors speculate that the MHCII peptide-binding groove requires N-glycans on its protein backbone to be sufficiently large for capturing glycoantigens, suggesting direct MHCII N-glycan involvement in glycoantigen binding (Ryan et al., 2011). MHCII N-glycosylation seems to be very different depending on the type of antigen-presenting cell, which could fine-tune the information exchange with T cells (Ryan and Cobb, 2012).

### Immune Signaling

Signaling immune receptors on B and T lymphocytes are also glycosylation-dependent. The receptor of the proinflammatory cytokine IFNγ (IFNγR) is composed of two IFNγR1 and two IFNγR2 subunits. In several children suffering from hypersensitivity to non-tuberculous mycobacterial infections, a homozygous T168N mutation is present in the IFNγR2 extracellular domain, creating an additional N-glycosylation site (Vogt et al., 2005; Moncada-Velez et al., 2013). This additional N-glycan negatively affects folding leading to slightly decreased IFNγR expression levels (Moncada-Velez et al., 2013). In contrast to wild-type IFNγR2, which is localized in lipid nanodomains, T168N mutants that do reach the cell surface are confined by the cortical actin meshwork (Blouin et al., 2016). Upon IFNγ binding, the wild-type receptor undergoes a conformational change resulting in JAK activation. However, the actin meshwork prevents IFNγR2-T168N to change conformation thus blocking IFNγ signaling. In line, galectin depletion rescues the T168N phenotype and galectin addition to wild-type IFNγR2 impaired IFNγ signaling (Blouin et al., 2016). These studies show that not only glycan loss or truncation can lead to receptor malfunction but that also additional receptor glycosylation sites may lead to disease.

Glycan-galectin interactions are important for many more receptors, including the T cell receptor (TCR). T cells from mice deficient in the glycosyltransferase GnT-V show a lack of complex branching N-glycans, which reduces Galectin-3 binding and enhances TCR clustering, resulting in autoimmunity (Demetriou et al., 2001; Chen et al., 2007). On B cells, BCR signaling is influenced by Galectin-9 dependent N-glycan mediated lateral interactions between the BCR and CD22, an ITIM bearing member of the sialic acid-binding immunoglobulin-like lectin (Siglec) family (Wasim et al., 2019). CD22 is heavily glycosylated and forms homo-oligomers by binding glycans of neighboring CD22 molecules in cis on the B cell membrane (Han et al., 2005). CD22 function as inhibitor of BCR signaling is hampered by removing five of its 12 glycosylation sites (Wasim et al., 2019). These glycans on CD22 may be bound by Galectin-9 and crosslinked with the BCR, thus explaining the inhibitory function of CD22 and Galectin-9 on BCR signaling (Cao et al., 2018; Wasim et al., 2019).

### Adhesion and Migration

Dynamic leukocyte-leukocyte and leukocyte-matrix interactions shape immunity and depend on many different adhesion receptors, including cadherins, selectins, and integrins. Integrins form a big family of heavily glycosylated transmembrane α/β heterodimeric proteins (Marsico et al., 2018). Although most integrin glycosylation studies focus on cancer cells or fibroblasts, integrin-mediated adhesion and migration is also key to elicit effective immune responses. Adhesion of the fibronectin-binding integrin α5β1 during myeloid differentiation into macrophages, e.g., is regulated via phorbol myristate acetate (PMA)-induced reduction of sialyltransferase ST6GAL1 activity, which causes β1-integrin hyposialylation and increased fibronectin binding (Semel et al., 2002). In fact, complex integrin glycosylation is required for heterodimerization, clustering, activation as well as lateral interactions with other membrane proteins (Zheng et al., 1994; Guo et al., 2002; Isaji et al., 2006, 2009; Hou et al., 2016). Leukocyte-specific β2-integrins are also highly glycosylated (Miller and Springer, 1987), and interactions between αLβ2 and galectins on human monocytes and dendritic cells are revealed by proteomics (Eich et al., 2016). However, information on glycan-mediated functional consequences is lacking. The β2-integrin ligands ICAM-1 and JAM-A on the endothelium carry N-glycans that are only marginally important for protein transport to the membrane but strongly influence protein conformation, dimerization, and binding to αLβ2 (Jimenez et al., 2005; Scott et al., 2015). Recently, two distinct N-glycoforms of ICAM-1 have been detected on activated endothelial cells: a complex, highly abundant N-glycoform and a less abundant oligomannose glycoform. These differ in their signaling capacity and dynamic interactions with the cortical actin cytoskeleton, possibly modulating distinct aspects of the inflammatory response (Scott et al., 2013).

Considering the importance of integrins and their ligands in many aspects of immune cell function, their glycan micro- and macroheterogeneity could be additional regulatory mechanisms of immune cell functions for which further investigation is warranted.

## Quantitative and Qualitative Measurement of Receptor Glycosylation: Chasing a Moving Target

To determine functional differences of receptor glycoforms, one needs methodologies (Table 1) that determine localization and site-occupancy of glycosylation sites, structure of glycan chains, glycan micro- and macroheterogeneity and glycoform-specific localization and interactome at the cell membrane. Ideally, these methodologies should also be able to capture the dynamic changes in these parameters.

First insights into the glycosylation status of immune receptors can be obtained by size-separating proteins using SDS-PAGE and immuno-detection of the protein of interest, before and after treatment with glycan-cleaving enzymes (Freeze and Kranz, 2010). Glycan removal reduces the molecular weight and results in a mobility shift of the protein. To obtain information about glycan composition, the so-called lectin blotting can be used on the intact glycoproteins (Sato, 2014). Recombinant lectins can also be used to label glycans present on the membrane of intact cells for detection by flow cytometry or microscopy (Bull et al., 2015), but their multimeric nature and relatively weak binding strength (Debray et al., 1981) may complicate data interpretation. Antibodies against glycan antigens are limited due to glycan low immunogenicity and high structural similarity (Tommasone et al., 2019). Another way to label membrane-exposed glycan structures on intact cells or in cell lysates is chemoenzymatic labeling, which exploits engineered recombinant glycosyltranferases that add monosaccharide analogs, suitable for click chemistry reactions, to specific structures in glycan chains (Lopez Aguilar et al., 2017). Although these techniques enable labeling of specific glycan structures, several limitations should be considered. It is plausible that glycocalyx thickness influences binding of lectins or access of glycosyltranferases and this can complicate data interpretation when variations in the glycocalyx composition are expected, such as between cell types, different metabolic states or in disease. Furthermore, staining and labeling methods provide ensemble measurements, thus lacking information about glycan microheterogeneity, and do not focus on a particular receptor of interest.

A solution may come from the combined use of a receptor-specific antibody and a glycan-specific probe, both adapted to serve in the so-called *in situ* proximity ligation assay (PLA) (Soderberg et al., 2006). The resulting co-detection of protein and glycosylation on the cell surface (Conze et al., 2010) has been successfully employed to demonstrate increased Sialyl Lewis X glycosylation of the RON receptor tyrosine kinase in cells overexpressing α2,3-sialyltransferase (Mereiter et al., 2016) and to reveal aberrant glycan modification of E-cadherin in human gastric carcinoma cells (Carvalho et al., 2016). However, since the distance between the two detected entities can be up to 40 nm, signals represent close proximity but may not reflect intramolecular co-occurrence (Alsemarz et al., 2018).

A technique that shows great potential for studying spatiotemporal organization of receptor glycoforms is the cis-membrane Förster resonance energy transfer (FRET)-based method for protein-specific imaging of cell surface glycans (Lin et al., 2014). This method introduces a FRET acceptor onto a specific monosaccharide of protein glycans, by metabolic labeling, and additionally a FRET donor onto the receptor of interest. FRET signals therefore solely originate from the labeled monosaccharide-containing protein of interest. Currently sialic acids and GalNAc residues can be labeled on membrane receptors in this way (Lin et al., 2014; Yuan et al., 2018) but future expansion of the monosaccharide toolbox will hopefully allow metabolic labeling of any sugar moiety on the glycoprotein of interest.

The techniques discussed above are of great value to detect glycoforms on the membrane, but do not provide detailed structural information about the glycan chains. To obtain this information, mass spectrometry can be used in a glycomics or glycoproteomics approach (Lauc and Wuhrer, 2017; Yang et al., 2017; Narimatsu H. et al., 2018). Although glycomics provides detailed structural information about the glycan chain without protein information and is therefore not suitable to study glycan position on receptors. For this, glycoproteomics is the preferred approach. Tryptic digestion of immunopurified glycoprotein receptors is required to generate glycosylated peptides for analysis by liquid chromatography-mass spectrometry (LC-MS). MS-based glycopeptide analysis is challenging since ionization efficiencies are much lower as compared to non-glycosylated peptides. In addition, the existence of multiple glycoforms of the same peptide reduces the abundance of the individual glycopeptide isoforms. Therefore, glycopeptides are commonly first enriched, e.g., via solid-phase extraction on polar cartridges. Recent advances further improve analytical sensitivity for glycopeptides (Narimatsu H. et al., 2018). Fragmentation of glycopeptides by tandem mass spectrometry (MS/MS) yields information on the glycan and peptide sequence to provide site-specific information on glycan heterogeneity (Stavenhagen et al., 2017).

A combination of labeling techniques to detect specific glycoforms and structural analysis of glycan composition and site-occupancy would greatly benefit our understanding of membrane receptor glycosylation.

## Strategies For Manipulation of Membrane Receptor Glycosylation

To study the function of glycan chains on membrane receptors, tools are needed to manipulate glycan site-occupancy and composition in a well-controlled manner. Thus far, the tools are rather generic. Metabolic glycosylation inhibitors, e.g., can block transport of proteins from the Golgi to the ER or interfere with the synthesis of nucleotide sugars, which are the building block of the lipid-linked oligosaccharide. Naturally, occurring glycan chain elongation inhibitors (e.g., plant alkaloids) block glycosylation enzymes and glycoside primers that mimic the natural glycosyltranferase substrates can divert glycan chain synthesis from the endogenous substrate (Wojtowicz et al., 2012; Esko et al., 2017).

The most widely used glycosylation inhibitor is the antibiotic tunicamycin, that inhibits GlcNAc phosphotransferase responsible for the initial steps in N-glycosylation, causing protein misfolding (Foufelle and Fromenty, 2016). Other inhibitors interfere with the enzyme oligosaccharyltransferase that is responsible for the transfer of the lipid-linked oligosaccharide to the protein (Lopez-Sambrooks et al., 2016; Rinis et al., 2018). Also, the development of specific glycosyltransferase inhibitors is pursued (Tedaldi and Wagner, 2014). Inhibitors, however, can have additional effects on general cellular functions, inducing ER stress and general toxicity, limiting their application.

Another way to manipulate protein glycosylation is through genetic means. The role of α1,3-fucosyltransferases and α(2,3)sialyltransferases on leukocyte rolling, e.g., was studied using shRNA-mediated knock-down (Buffone et al., 2013; Mondal et al., 2015). CRISPR/Cas9-mediated gene editing allowed the simultaneous determination of N-glycan, O-glycan and glycosphingolipid contributions to leukocyte rolling and adhesion (Stolfa et al., 2016). Recently, a gRNA library for CRISPR/Cas9 knockout of 186 glycosyltransferases has been validated in HEK293T cells (Narimatsu Y. et al., 2018), and its application to immune cell biology is an exciting opportunity.

The most specific way to manipulate the glycan content of a given receptor remains site-directed mutagenesis. The substitute amino acid should be carefully chosen, since it may affect protein structure independently from the loss/gain of the glycosylation site (Bordo and Argos, 1991). Moreover, expression of the mutant in presence of the endogenous protein should be avoided. This can be achieved by exploiting CRISPR/Cas9-triggered homology-directed repair in cells to introduce the glycosite mutation in the immune-receptor gene. Unfortunately, this technique allows only the loss or gain of complete glycans and not their editing. Further development of tools for targeted modification of receptor glycosylation and for dedicated assessment of glycan structure and site-occupancy is required.

## Outlook

Pioneering studies combining super-resolution microscopy with bioorthogonal chemistry have recently provided the first nanoscale images of membrane glycans and measurements of glycocalyx height differences (Letschert et al., 2014; Mockl et al., 2019). Since each methodology for glycan quantification and editing has limitations, smart combinations of techniques may be the way forward to reveal novel aspects of membrane glycan biology.

## Author Contributions

AC and PH designed and wrote the review with input from WH and DL for conceiving, writing, and editing the manuscript.

### Conflict of Interest

The authors declare that the research was conducted in the absence of any commercial or financial relationships that could be construed as a potential conflict of interest.
